# Cancer Metabolism as a New Real Target in Tumor Therapy

**DOI:** 10.3390/cells10061393

**Published:** 2021-06-05

**Authors:** Ferdinando Chiaradonna, Domenica Scumaci

**Affiliations:** 1 Department of Biotechnology and Biosciences, University of Milano Bicocca, 20126 Milano, Italy; 2 Research Center on Advanced Biochemistry and Molecular Biology, Department of Experimental and Clinical Medicine, Magna Græcia University of Catanzaro, 88100 Catanzaro, Italy

## 1. Introduction

Cancer cells exhibit common hallmarks consisting of specific competencies acquired during the tumorigenesis process, including stimulation of cancer cell proliferation, insensitivity to growth signal inhibition, apoptosis evasion, enhancement of replicative potential, induction of angiogenesis, and tissue invasion and metastasis. To sustain the high rate of proliferation, most cancer cells use metabolic adaptations promoting their survival under harsh conditions. These phenomena are known as metabolic rewiring. Metabolic rewiring is considered the major pathogenic driving force for malignant transformation. In cell transformation, arising in different cell types through the dysregulation of several pathways, metabolic rewiring is a common feature founded on three main requirements: ATP generation, synthesis of biological precursors for sustaining cell growth, and efficient handling of reactive oxygen species (ROS) for the maintenance of the oxidative balance. More recently, cancer metabolic alterations have been shown to play a role in the sensitivity of cancer cells to the most used chemotherapeutics, suggesting that cancer metabolic rewiring is an important mediator of drug-resistant cancer [1,2,3,4,5].

Cancer cellular rewiring is the result of an intricate network of classic cancer metabolic pathways and a number of emerging dysregulated pathways supporting cell proliferation including increased uptake of nutrients, enhanced glycolysis, enhanced shunt of pentose phosphate, glutaminolysis, fatty acid synthesis and degradation, stimulation of autophagy, micropinocytosis, and gene expression alterations depending on histone metabolic changes.

The redundancy and overlapping of several of these networks account for plasticity that is crucial for cancer cell survival.

More evidence is also suggesting that the tumor microenvironment plays a key role in metabolic rewiring, indicating that the understanding of the interaction between specific tumor-intrinsic mechanisms and cell-extrinsic stimuli might elucidate the pathways that lead to immune evasion [6,7].

In summary, although our understanding of cancer metabolic rewiring has considerably progressed, much remains to be learned to better understand tumor biology and improve therapeutic approaches. Indeed, the elucidation of pathways underlying metabolic reprogramming will have a tremendous impact by shedding light on therapeutic targets useful for developing new translational clinical approaches. From this perspective, the purpose of this Special Issue is to gather reports further defining previous and/or newly discovered metabolic alterations associated with oncogenesis and their functional contribution to the establishment and maintenance of tumor phenotypes as well as papers reporting the potential druggability of metabolic pathways, alone or in combination with other currently used chemotherapeutics, to improve cancer therapy.

The Special Issue “Metabolic Rewiring in Cancer Cells: From Tumor Onset to Novel Therapies”, guested by Ferdinando Chiaradonna and Domenica Scumaci, attracted a total of 28 regular papers, spanning over numerous old-fashioned and emerging topic areas in the field of cancer metabolism. Since the Special Issue aimed to give an overview of recent studies on cancer metabolism, it hosts 13 original reviews and 15 original research articles.

## 2. Synopsis

From the seminal observation of Otto Warburg regarding the cancer-specific energetic requirement to enhance glycolysis over oxidative phosphorylation (OXPHOS), also under normoxia, several findings have expanded the glycolytic role in cancer metabolic rewiring and growth as well as the number of proteins involved in glycolysis regulation [8,9,10].

In this regard, Zonta and colleagues [11], by means of selective depletion of Casein Kinase 2 (CK2) catalytic subunits, demonstrated the (α) CK2 catalytic subunit contributes to the glycolytic phenotype of neuroblastoma and osteosarcoma cancer cell lines, favoring cell proliferation, survival, and tumorigenicity. This concept was also underlined by Venturoli and colleagues [12], who showed in mice xenografts, derived from the highly glycolytic OC316 and OVCAR3 ovarian cancer cell lines in which PDK1 is silenced, a significant change in their metabolic features, i.e., an increased OXPHOS, associated with a significant reduction in tumor growth and angiogenesis. On the other hand, Ambrosini and colleagues [13], by using a model of pancreatic cancer stem cells (CSCs), also showed that a metabolic shift from glycolysis to OXPHOS causes a quiescent state, suggesting an association between proliferation and glycolysis also in CSCs. A further glycolytic role in tumor aggressiveness was suggested by Miranda-Gonçalves and colleagues [14]. Indeed, they delineated a new mechanism that is SIRT1-mediated by which lactate contributes to renal cancer cell aggressiveness and pseudo-transformation of adjacent normal cells. They postulated that excreted lactate by glycolytic cancer cells inhibits SIRT1 in adjacent tumor and normal cells, causing histone H3 hyperacetylation and tumor cell aggressiveness. A link between histone modifications and glycolysis was also described by Scumaci and colleagues [15]. In particular, they defined a histone protein modification, namely, advanced glycation end products (AGE), due to the Warburg effect. Importantly, this process that causes histone code loss appears consequent to inactivation of DJ1, involved in the selective removal of AGE from histones.

A common feature of glycolytic solid cancer is hypoxia, whose master regulator, hypoxia inducible factor (HIF), orchestrates and triggers prosurvival and prometastatic events. In this context, the review of Belisario and colleagues [16] offers a clear and comprehensive synopsis of how peculiar hypoxia-related events cooperate in determining chemoresistance, hence suggesting an intriguing starting point for the development of novel therapeutic strategies.

Since designing new anticancer therapies is an important goal of the fight against cancer, metabolic rewiring is becoming an important subject of study to pave new roads either in terms of novel therapy or identification of chemoresistance mechanisms. In this regard, several manuscripts in this Special Issue address this point. Lorito and colleagues [17] described the different metabolic adaptation of ER+/HER2+ and ER+/HER2− breast cancer cells upon palbociclib (Cdk4/6 inhibitor) treatment. In fact, while the former cells become palbociclib-resistant in tight association with an enhancement of glycolysis, the latter become resistant in tight association with an enhancement of OXPHOS, suggesting that metabolic targeting could represent a good therapeutic option in both types of patients. Glucose metabolism as a possible target was also addressed by Ricciardiello and colleagues [18]. Indeed, by using different pancreatic cancer cell models, they showed that inhibition of HBP, a downstream glycolytic branch, induces cell proliferation arrest and death in an oncogenic K-Ras-dependent fashion. In addition, they showed that HBP activation is also associated with resistance to a pan-ras inhibitor, suggesting that glycolysis and downstream pathways are tightly linked to chemoresistance. In the context of an unconventional source for the glycolysis pathway, the review of Krause and colleagues [19] discussed how cancer cells may use fructose as an alternative glycolytic substrate as well as for lipogenesis and nucleotide synthesis. They focused on the link between fructose metabolism and cancer cell proliferation, exploring how fructose is metabolized in different types of cancer and how it associates with cancer development and outcome.

Conversely, Albanesi and colleagues [20] found that ATRA treatment, a differentiation therapy, in the acute promyelocytic leukemia cell line NB4, correlates with the activation of aerobic glycolysis and the reduction in OXPHOS-dependent ATP production, proposing that granulocytic differentiation and patient therapy could be favored by OXPHOS inhibition. A tight association between current cancer therapies and metabolic rewiring was also proposed by Verma and colleagues [21]. Indeed, they showed that prolonged enzalutamide treatment of the prostate cancer cell lines LNCaP and C4-2B induces resistance by enhancing the gene expression of solute carrier genes. Latter causes dysregulation of glucose, fatty acid, and lipid metabolism pathways, suggesting a central role of solute carrier genes during metabolic reprogramming of prostate cancer cells in an androgen-deprived environment. The therapeutic targeting of solute carriers was well addressed in the review of Scalise and colleagues [22] that summarizes current knowledge on the topic. They offer a critical point of view of the literature on inherent solute carriers that are over-expressed in human cancers and that represent an appealing target for anticancer drugs.

Fatty acid (FA) metabolism rewiring in cancer upon the onset of chemoresistance was described in the manuscript of Brindisi and colleagues [23]. Indeed, they showed that exogenous cholesterol and mevalonate induce breast cancer cell proliferation, aggressiveness, and drug resistance, through the activation of the estrogen-related receptor alpha (ERRα) pathway that is eventually responsible for intense metabolic switching, higher proliferation rates, sustained motility, propagation of CSCs, and lipid droplet formation. These findings open the possibility to target the cholesterol/mevalonate/ERRα axis as a new potential therapy in breast cancer. The role of exogenous FA in breast cancer cell aggressiveness is also the focus of the manuscript from Rizzo and colleagues [24]. Indeed, they showed a different sensitivity of MDA-MB-231 and MCF7 breast cancer cells to lipid microenvironment changes. In particular, exogenous FA addition (i.e., palmitic and docosahexaenoic acids) perturbs the ER membrane architecture and desaturase activity, especially in MDA-MB-231 cells, suggesting that the lipid microenvironment could influence cell malignancy and chemoresistance onset.

Metabolic rewiring as a target to eradicate cancer was well addressed by Fiorillo and colleagues too [25]. Indeed, they showed that deferiprone, a clinically approved iron chelator, can effectively target mitochondria in CSCs derived from ER+ MCF7 and T47D cells, causing a significant reduction in OXPHOS and an accumulation of ROS that, together, lead to cancer cells’ inability to form 3D tumorspheres. Metabolic targeting in 3D models is also the focus of the manuscript from Pasquale and colleagues [26]. The authors, by using the two bladder cancer cell lines RT112 and 5637, showed that both cell lines have a mixed energetic phenotype since they are able to produce ATP equally well through OXPHOS and glycolysis. However, meanwhile, the two cell lines show a different metabolic plasticity, OXPHOS targeting by means of metformin, significantly affects spheroid formation of both cell lines, suggesting, again, that CSCs mitochondria could be an efficacy target to eradicate cancer. Another interesting metabolic therapeutic approach designed for non-small cell lung cancer (NSCLC) is provided by Caiola and colleagues [27]. The authors, by using a metabolomics approach, defined a compensative metabolic pathway active in NSCLC cells upon treatment with the glutaminase inhibitor CB-839. Importantly they demonstrated that the different sensitivity to CB-839 of NSCLC cells is not dependent on KRAS mutations or LKB1 loss but on the ability of cancer cells to use external alanine to increase pyruvate synthesis and the TCA cycle. Consequently, they showed that inhibition of alanine catabolism, by using l-cycloserine (a GPT2 inhibitor), negatively affects NSCLC CB-839-resistant cell growth and proliferation.

Recently, the role of micronutrients, as a determining factor in cancer metabolism adaptation, is becoming an interesting issue. In this context, dysregulation of iron metabolism has been related to several types of cancer, being implicated in ROS production, ferroptosis, and malignant transformation [28]. In our Special Issue, three interesting reviews focused on iron metabolism, underlining several aspects of this research area. Hsu and colleagues [29] offer a cover of the main iron metabolism alterations that impact tumorigenesis as well as tumor growth. In a translational perspective, the authors remarked the necessity to develop iron-targeted anticancer therapy with a special emphasis on the tumor microenvironment. Similarly, Sacco and colleagues [30] proposed a good synopsis of crucial and controversial aspects of iron homeostasis together with an overview on the current anticancer therapeutic strategies based on iron targeting.

On the other hand, Di Sanzo and colleagues [31] summarized current knowledge on ferritin pseudogenes, discussing the importance of unraveling their molecular functions in the context of the oncogenic network.

Iatrogenic targeting of tumor susceptibilities is also a promising area of research. Although Warburg theory postulated that the dependence of cancer cells on glycolysis was due to their malfunctioning mitochondrial respiration, recently, it became clear that functional mitochondria are a key cancer cell feature [32,33]. In this scenario, mitochondria represent a vulnerable target for the development of therapeutic approaches. In our Special Issue, we hosted several reviews addressing this point. Fu and colleagues [34] discussed recent knowledge on the implementation of strategies to target mitochondria and overcome chemoresistance in pancreatic cancer; additionally, Audano and colleagues [35] reviewed the main pathways involved in the restricting mitochondrial checkpoint that leads to tumor transformation. Lastly, Cioce and colleagues [36] proposed an overview of the use of metformin as a key anticancer molecule targeting mitochondria. Notably, they provided a special paragraph on the effects of metformin on CSCs whose plasticity accounts for chemoresistance, relapse, and metastasis.

Metastatic progression is based on the ability of cancer cells to acquire an adaptive phenotype disseminating to distant organ sites and evading the immune system. In this context, two comprehensive reviews, proposed by Roda and colleagues [37] and by Benzarti and colleagues [38], examined complementary features of this critical matter, pointing out the ability of cancer cells to gain a bioenergetic shift and discussing the role of metabolic reprogramming in eliciting the metastatization process.

During cancer growth and metastatization, immune system evasion is a crucial and determinant event. Pellegrino and colleagues [39] discussed this topic in a therapeutic perspective exploring recent strategies aiming at boosting metabolic CAR T-cell fitness to enhance their anti-tumor activity.

Finally, in the field of drug discovery, we would also like to underline the manuscript from Nardone and colleagues [40] that, by using experimental and computational approaches, suggests the use of suramine or its analogues to inhibit the transcription factor NF-Y, often over-expressed in cancer, and a key factor in the control of genes involved in nucleic acid, amino acid, glucose, and lipid metabolic pathways and therefore directly involved in cancer metabolic rewiring. Finally, we would like to highlight the review of Ferraro and colleagues [41] that summarizes current knowledge on the use of “Multi-Target Ruthenium (III) Complex Lodged in Nucleolipid Nanosystems” for the treatment of breast cancer.

## 3. Conclusions

This Special Issue widely describes several metabolic alterations of cancer cells favoring fast proliferation, migration, invasion, and CSCs survival. In addition, given the fundamental role of cancer metabolic rewiring in cancer progression and chemotherapy resistance, several metabolic approaches, interfering with glycolysis, OXPHOS, fatty acid metabolism, and glutaminolysis, were presented. These strategies could represent a novel class of therapeutic approaches that, alone or in combination, will improve current cancer therapies.
